# Access to In-Network Emergency Physicians and Emergency Departments Within Federally Qualified Health Plans in 2015

**DOI:** 10.5811/westjem.2015.12.29188

**Published:** 2016-01-20

**Authors:** Stephen C. Dorner, Carlos A. Camargo, Jeremiah D. Schuur, Ali S. Raja

**Affiliations:** * Vanderbilt University School of Medicine, Department of Emergency Medicine, Nashville, Tennessee; † Harvard T.H. Chan School of Public Health, Department of Health Policy & Management, Boston, Massachusetts; ‡ Massachusetts General Hospital, Department of Emergency Medicine, Boston, Massachusetts; § Brigham and Women’s Hospital, Harvard Medical School, Department of Emergency Medicine, Boston, Massachusetts

## Abstract

**Introduction:**

Under regulations established by the Affordable Care Act, insurance plans must meet minimum standards in order to be sold through the federal Marketplace. These standards to become a qualified health plan (QHP) include maintaining a provider network sufficient to assure access to services. However, the complexity of emergency physician (EP) employment practices – in which the EPs frequently serve as independent contractors of emergency departments, independently establish insurance contracts, etc… – and regulations governing insurance repayment may hinder the application of network adequacy standards to emergency medicine. As such, we hypothesized the existence of QHPs without in-network access to EPs. The objective is to identify whether there are QHPs without in-network access to EPs using information available through the federal Marketplace and publicly available provider directories.

**Results:**

In a national sample of Marketplace plans, we found that one in five provider networks lacks identifiable in-network EPs. QHPs lacking EPs spanned nearly half (44%) of the 34 states using the federal Marketplace.

**Conclusion:**

Our data suggest that the present regulatory framework governing network adequacy is not generalizable to emergency care, representing a missed opportunity to protect patient access to in-network physicians. These findings and the current regulations governing insurance payment to EPs dis-incentivize the creation of adequate physician networks, incentivize the practice of balance billing, and shift the cost burden to patients.

## INTRODUCTION

The Emergency Medical Treatment and Active Labor Act (EMTALA), passed in 1986, guaranteed access to emergency medical care.^1^ However, it did not guarantee that health insurers would pay for that care. As a result, patients seeking care from emergency departments (EDs) outside of their insurance networks commonly faced higher out-of-pocket charges (cost-sharing) in the form of co-insurance and co-payments than from EDs in their insurance networks. The Affordable Care Act (ACA) attempted to eliminate that practice and standardize those out-of-pocket costs by prohibiting insurance companies from imposing higher cost-sharing at out-of-network EDs than what is required at in-network EDs.^2^ Recently, however, the issue of high-cost emergency care and “surprise medical bills” have re-emerged as a result of “balance billing,” where patients are billed by out-of-network emergency physicians (EPs) at in-network facilities.^3^ The outcry against this practice has prompted states to evaluate means to improve consumer protections, including bans on balance billing.

One potential protection already in place is the federal regulation of qualified health plans (QHPs). The ACA granted the U.S. Department of Health and Human Services (HHS) authority to establish federal network adequacy standards for plans certified for sale through the Health Insurance Marketplace. Those QHPs must “maintain a network that is sufficient in number and type of providers… to assure that all services will be accessible without unreasonable delay.”^4^ Thereafter, network adequacy is enforced under a “reasonable access” standard that provides leniency for the structure of insurers’ provider networks.^5^

Although the HHS network adequacy standards are generalizable to all medical specialties, their applicability to emergency medicine is unclear given the unique regulatory space the field occupies. The template submitted by carriers to HHS for QHP certification requests the number of providers in particular specialties but does not include emergency medicine among them.^6^ Additionally, despite the submission of that information prior to QHP certification, a recent study using provider directory information found an alarming number of QHPs offered in 2015 lacked access to in-network physicians for multiple specialties.^7^ In light of this finding, and because network design is a potential driver of balance billing, we sought to determine how well the existing network adequacy standards protect access to in-network emergency care.

## METHODS

We performed an analysis of QHP provider networks using previously published methods,^7^ examining physician networks in the 34 states participating in the federal Marketplace during 2015 open enrollment. We used each plan’s federally mandated public provider directory to assess access to EPs and hospitals within each QHP’s network. In a previous study, provider directory findings were verified with phone calls to carriers.^7^ Federal regulations require QHP issuers to make provider directories publicly available to allow consumers “to view the provider network that is specific to a given QHP.”^4^ As such, provider directories are expected to be an appropriate tool for assessing consumers’ access to in-network care.

We conducted our search between April 11, 2015, and April 12, 2015. In the rating area (the geographic unit for Marketplace premiums) containing the most populous county within each state, we analyzed four silver plans, the most popular plans purchased by 69% of consumers;^8^ these include the lowest, second-lowest, median, and highest premium plans. The second-lowest premium plan was analyzed because federal subsidies are tied to those particular plans. To conservatively account for patient travel, we applied a search radius of 50 miles relative to the primary U.S. postal service zip code pertaining to each rating area’s most populous city. Our chief outcome of interest was identifying whether QHPs had in-network EPs and hospitals.

## RESULTS

We analyzed a total of 136 silver QHPs. Among them, we identified 30 QHPs (22%) with provider networks completely lacking identifiable EP coverage (Figure). Uniformly, the provider directories for all 136 plans provided the functionality to search for physicians by specialty. However, 16 QHPs (11.8%) did not list emergency medicine as a searchable specialty.

Five QHPs (3.7%) lacked hospital coverage. Among them, three QHPs (2.2%) covered EPs but did not cover a hospital and two QHPs (1.5%) lacked both in-network EP and hospital coverage. Compared to previously published data regarding QHPs without access to in-network specialists, there are substantially more provider networks lacking EP coverage (22%) than for any other specialty analyzed (Table).

QHPs lacking EPs spanned 15 (44%) of the 34 states using the federal Marketplace. Importantly, information regarding whether EPs were hospital employees or independent contractors was commonly not available within the provider directories.

## DISCUSSION

One in five provider networks in a national sample of Marketplace plans lacks identifiable in-network EPs. Though this does not necessarily entail a lack of access to emergency care, it represents an apparent lack of access to insured emergency care. Additionally, the wide disparity between access to in-network hospitals and in-network EPs suggests the potential for balance billing.

While many patients may assume that when they go to the ED at an in-network hospital they will be treated by hospital employees, that is most commonly not the case. Only 21.2% of EPs are hospital employees.^9^ The majority of EPs are employed by physician groups, which negotiate their own insurance contracts and staff EDs with independent contractors. A 2014 analysis found that within the network for one of the largest insurance carriers in Texas, more than half of the hospitals were not staffed with any in-network EPs.^10^ Our data suggest such a scenario is common, creating widespread potential for balance billing. Just how frequently the practice occurs is unclear, but only one-fourth of states currently have some form of consumer protection against bills from out-of-network providers.^11^

In an attempt to ensure that QHPs do not pay unreasonably low amounts to out-of-network EPs, the ACA specified a minimum reimbursement threshold for emergency care provided out-of-network.^12^ Carriers must pay out-of-network providers the greatest of the plan’s median payment amount for in-network providers, a payment based on the usual methods the plan uses to determine payments for other out-of-network services, or the amount that Medicare would pay for those services. However, EPs report that these payment thresholds are insufficient to cover the cost of emergency care.^13^ As such, EPs are incentivized to balance bill, and patients subsequently carry a greater cost burden.

## LIMITATIONS

This study disproportionately analyzes lower-cost plans. However, network-adequacy standards are uniform and do not differ by premium pricing. Additionally, our results may be due to issues with the transparency of provider directories themselves. The template submitted to HHS for QHP certification does not contain the full breadth of information available in the provider directories. For 2015 QHPs, HHS required an “up-to-date provider directory where the consumer can view the provider network that is specific to a given QHP.”^4^ Emergency medicine is not specifically identified in the QHP certification submission; however, the provider directories are required to be “complete.” As such, our data may be due to a lack of provider directory transparency.

However, 21% of plans in our sample list 1–5 in-network EPs, suggesting that at least these plans identify EPs; there is clearly no blanket policy excluding EPs from directory inclusion. Instead, this suggests that our results are driven by network inadequacy rather than directory structure. Furthermore, although there have been problems with provider directories, the predominant issue has been an over-reporting of in-network providers. Thus, under the presumption that carriers were compliant in publishing an up-to-date directory of the provider network, the in-network physicians should be identifiable by any specialty. Even a perceived lack of access to a specialty could disrupt the market, and consumers should be empowered to make informed market choices. In the interest of transparency, HHS’ final letter to 2015 issuers indicated that provider directories should “include location, contact information, specialty, and medical group, any institutional affiliations for each provider, and whether the provider is accepting new patients.”^4^ We did not find this to be common practice. HHS has imposed more stringent regulations on provider directories for QHPs offered in 2016 to ensure greater accuracy.^14^,^15^

## CONCLUSION

Our findings suggest that there is an unfulfilled opportunity to apply network adequacy standards to emergency medicine and ensure sufficient access to in-network emergency care. Without requiring QHPs to submit in-network EPs as part of the network adequacy template, it is impossible to provide oversight for adequate in-network access to emergency care. In the absence of that enforcement, insurance companies are incentivized to pay the minimum repayments outlined by the ACA to out-of-network providers, and EPs are consequently incentivized to balance bill their patients. As states seek to ban balance billing, in the absence of meaningful network adequacy or payment reform, EDs will be hard pressed to recuperate the costs of providing emergency care.

Ultimately, the most effective way to protect affordable patient access to emergency care may be to enact “Any Emergency Physician” regulations requiring that QHPs effectively view all EPs as “in network” and negotiate reasonable reimbursements rather than attempting to enforce, determine, and regulate the sufficient number of physicians needed to provide adequate in-network access to emergency care.

## Figures and Tables

**Figure:**
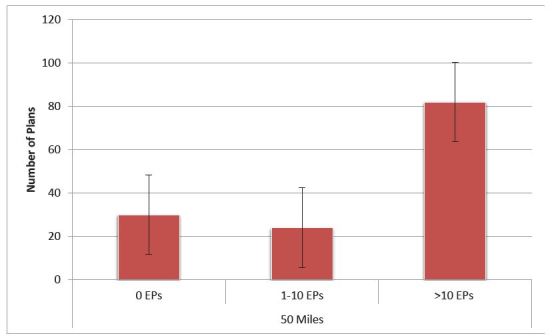
Number of Affordable Care Act plans with in-network emergency physicians by number of physicians and search radius. Sample contained 136 plans in the Silver tier on the federal Marketplace in 2015, representing the lowest, second-lowest, median, and highest premium plans in each of the 34 federal Marketplace states. Search radius was defined as 50 miles for most plans; for the 2% of plans that did not offer that radius for their provider network search tool, we used the maximum available search radius (typically 25–35 miles). *EPs*, emergency physicians

**Table:** In-network physicians by specialty within 50 miles. Sample contained 136 plans in the Silver tier on the federal Marketplace in 2015, representing the lowest, second-lowest, median, and highest premium plans in each of the 34 federal Marketplace states. Search radius was defined as 50 miles for most plans; for the 2% of plans that did not offer that radius for their provider network search tool, we used the maximum available search radius (typically 25–35 miles). Percentages are expressed as out of 136 plans for emergency medicine and 135 plans for all other specialties due to defective provider directories.

Specialty	Number of plans with 0 specialists (%)	Number of plans with 1–2 specialists (%)	Number of plans with 3–5 specialists (%)	Number of plans with >5 specialists (%)
Cardiology	1 (0.7)	2 (1.5)	0	132 (97.8)
Dermatology	5 (3.7)	0	0	130 (96.3)
Emergency medicine	30 (22)	8 (5.9)	13 (9.6)	85 (62.5)
Endocrinology	11 (8.0)	0	3 (2.2)	121 (89.6)
Neurology	1 (0.7)	1 (0.7)	0	133 (98.5)
OB/GYN	2 (1.5)	0	0	133 (98.5)
Oncology	2 (1.5)	0	7 (5.2)	126 (93.3)
Psychiatry	6 (4.4)	1 (0.7)	2 (1.5)	126 (93.3)
Pulmonology	1 (0.7)	2 (1.5)	6 (4.4)	117 (86.7)
Rheumatology	9 (6.7)	5 (3.7)	0	121 (89.6)

*OB/GYN,* obstetrics and gynecology
